# Adherence to the therapeutic guidelines recommendations among the people with type 2 diabetes mellitus and obesity, frailty, or recent diagnosis, attended in primary health care centers in Spain: A cross-sectional study

**DOI:** 10.3389/fmed.2023.1138956

**Published:** 2023-03-24

**Authors:** Bogdan Vlacho, Manel Mata-Cases, Berta Fernandez-Camins, Laura Romera Liébana, Joan Barrot-de la Puente, Josep Franch-Nadal

**Affiliations:** ^1^DAP-Cat Group, Unitat de Suport a la Recerca Barcelona, Fundació Institut Universitari per a la Recerca a l'Atenció Primària de Salut Jordi Gol i Gurina (IDIAPJGol), Barcelona, Spain; ^2^Institut de Recerca Hospital de la Santa Creu i Sant Pau, Barcelona, Spain; ^3^CIBER of Diabetes and Associated Metabolic Diseases (CIBERDEM, ID CB15/00071), Instituto de Salud Carlos III (ISCIII), Barcelona, Spain; ^4^Primary Health Care Center La Mina, Gerència d'Àmbit d'Atenció Primària Barcelona Ciutat, Institut Català de la Salut, Sant Adrià de Besòs, Spain; ^5^Primary Health Care Center Poblenou, Gerència d'Àmbit d'Atenció Primària Barcelona Ciutat, Institut Català de la Salut, Sant Adrià de Besòs, Spain; ^6^Primary Health Care Center Doctor Lluís Sayé, Gerència d'Àmbit d'Atenció Primària Barcelona Ciutat, Institut Català de la Salut, Barcelona, Spain; ^7^Center d'Atenció Primària Salt, Gerència d'Àmbit d'Atenció Primaria, Institut Català de la Salut, Girona, Spain; ^8^Primary Health Care Center Raval Sud, Gerència d'Àmbit d'Atenció Primària Barcelona Ciutat, Institut Català de la Salut, Barcelona, Spain

**Keywords:** clinical guideline adherence, primary care (PC), Spain, type 2 diabetes, antidiabetic drug

## Abstract

**Introduction:**

Clinical practice guidelines are helpful for clinicians, and their proper implementation could improve the quality of care and management of participants with diabetes. This study aimed to evaluate the degree of adherence to the Clinical Practice Guidelines (CPG) recommendations among obese, frail, or recently diagnosed type 2 diabetes mellitus (T2DM) participants in primary care centers in Spain.

**Methods:**

We perform a cross-sectional study on a national level in two phases. In the first phase, study participants were recruited, and their clinical data were collected. In the second phase, data related to the participating physicians were collected.

**Results:**

In total, 882 participants from 240 physicians were analyzed. According to the study questionnaire, most participants from all three clinical groups had adequate adherence to the CPG. This percentage was highest among the recently diagnosed T2DM (91.6%) and lowest percent of frail T2DM persons (74.7%). The inadequate adherence to the guidelines was observed mainly among the obese and frail participants with T2DM from medical doctors with low CPG knowledge (3.4% and 3.5%, respectively). Regarding the patient's characteristics and degree of adherence to the guidelines, the participants with inadequate adherence were generally older, with higher BMI, poorer HbA1c control, and fewer visits with primary care physicians. Most (57%) primary care physicians had moderate CPG knowledge. In our multivariable logistic model, we did not observe statistically significant odds ratios for different characteristics related to the physicians/consultation and low CPG knowledge.

**Discussion:**

The results of our cross-sectional study observe adequate adherence to the clinical guidelines by the primary care physicians for the majority of the participants with obesity, frailty, or newly diagnosed with T2DM.

## 1. Introduction

Type 2 diabetes mellitus (T2DM) is a highly prevalent chronic disease where proper control plays an important role in preventing damage to multiple organs or tissues. According to the International Diabetes Federation, it is expected that 1 in 10 adults will be affected by this disease until the year 2045 worldwide, when an especially high prevalence is expected among the population over 65 years of age (1). Currently, in Spain, the prevalence of T2DM is among the highest in Europe, with 14.8% (~5.1 million adults) and an incredible increase of 42% just in 2 years since 2019 (2).

Poor metabolic control is associated with increased complications and premature mortality and is the leading cause of blindness, renal replacement therapy (dialysis/transplantation), and non-traumatic amputations (3). Early and multifactorial treatment and interventions could delay the onset of complications and improve quality and life expectancy (4). The therapeutic approach to the disease should be multidisciplinary and include pharmacological and non-pharmacological strategies and measures for preventing comorbidities and long-term complications (5–7). According to the therapeutic guidelines, the pharmacological treatment should be established gradually in therapeutic steps and in an individualized manner, considering patient- factors, such as age, the presence of associated comorbidities, the degree of prior control, and the presence or absence of other concomitant diseases and treatments (5, 7, 8).

In recent years, the scientific and clinical evidence regarding diabetes treatment increased substantially. The growing number of diabetes treatments (behavioral interventions, pharmacological treatment, surgery) and increasing information about their benefits and risks offer more options for people with diabetes and their health providers. Only in Europe, from 2005 to 2017, the European Medicines Agency (EMA) approved 40 new drugs for treating diabetes (9). Evidence-based practice has evolved as a dominant way of practice, policy, management, and education within health services throughout the developed world. However, the vast amount of information could complicate the decision-making process for proper treatment selection. Clinical practice guidelines, and local clinical and therapeutic protocols, are documents that provide physicians with updated and structured information for better management of patients. Clinical and therapeutic guidelines are considered essential resources for planning, providing, evaluating, and improving the quality of healthcare services (10). The main aim of clinical guidelines is to improve and standardize the quality of care among patients. Although rigorous evaluations have shown that clinical practice guidelines can improve the quality of patient care in experimental settings (11), whether they achieve this goal in daily clinical practice is less clear. This issue could be since patients, clinicians, health providers/payers, and managers define quality differently, and current evidence on guideline effectiveness is incomplete.

Implementing clinical practice guidelines remains a significant challenge for health professionals. Evidence indicates that many patients do not receive adequate care and may even receive inadequate or potentially harmful care or therapy (12). The degree of therapeutic inertia in people with diabetes is high in our primary health care settings. In the recently reported analysis of Mata et al. on 301,144 people with diabetes, the authors found that antidiabetic treatments were not intensified in patients who were treated with two oral drugs, for 26.2% of people with HbA1c > 7% and 18.1% with HbA1c > 8%. Intensification with a third oral drug or insulin was performed with HbA1c of 8.7% and 9.4%, respectively (13). These results are similar to those found in the U.K., where treatment with oral antidiabetics or insulin with HbA1c of 9.1% and 9.7% took an average of 7 years to be intensified third antidiabetic drug (14). Another study in the U.K. evaluated adherence to the National Institute for Health and Care Excellence (NICE) guidelines for starting and continuing glucagon-like peptide 1 (GLP1) agonists in patients with T2DM. Only 25% of patients started GLP-1 receptor agonists as part of a NICE-recommended regimen (15). However, although health professionals can provide advice or recommendations about medications, food intake, and the effects of physical activity, the main factors determining the success, achievement, and maintenance of metabolic control are the patient's ability and willingness to self-management (16). In a qualitative study conducted by Berenguera et al. (17) in our primary care setting with poorly controlled T2DM patients, although disease control alone (self-management) should occur daily, it was often difficult due to family or financial reasons, lack of awareness, or lack of motivation.

Clinical practice guidelines are helpful for clinicians, and their proper implementation could improve the quality of care and management of participants with diabetes. However, as a clinical tool, the proper implementation and use should be evaluated to know their actual effect in the medical consultation. To the best of our knowledge, no studies have been conducted in our primary care settings to evaluate adherence to therapeutic guidelines for managing T2DM. The objective of this study was to evaluate the degree of adherence to the therapeutic recommendations of the Clinical Practice Guidelines (CPG) among participants with obesity, frailty, or recently diagnosed with T2DM in primary care centers in Spain.

## 2. Patients and methods

### 2.1. Study design and data source

We perform a cross-sectional study on a national level involving physicians from different primary care centers in Spain. The study was realized in two phases. In the first phase, study participants were recruited, and their clinical data were collected. In the second phase, data related to the participating physicians were collected. A specific survey was done to evaluate the degree of knowledge of the therapeutic guidelines, their opinion, and therapeutic preferences. The first phase was realized from June 1^st^, 2020, till November 30^th^, 2020 (6 months), while during December 2020 (1 month), we realized the second phase. The study recruitment consisted of offering the study participants to possible candidates. If participants agreed to enter the study, it was obligatory to sign the informed consent before any study procedure was performed or study data were collected. Regarding the primary care physicians selection, medical doctors from different regions of Spain were invited to participate in the study. If they agreed, they were involved in the study. All study procedures and data collection were performed in a single study visit. A contract research organization externally monitored the study recruitment and data collection.

### 2.2. Definition of eligibility criteria

T2DM (diagnosed according to the ADA—American Diabetes Association criteria) subjects aged at least 30 years old and with signed informed consent were included in the study. In order to identify the three groups of subjects: obesity, frailty, or recently diagnosed T2DM. We defined different sets of additional study criteria for each group. The T2DM subjects with obesity were requested to have ages between 55 and 74 years, a body mass index (BMI) of more than 30 kg/m^2^, a T2DM diabetes duration longer than 5 years, and treatment with at least one antidiabetic drug. The frail T2DM person had to be at least 75 years old, with a T2DM duration longer than 5 years, and on treatment with at least two antidiabetic drugs. The frailty was defined according to the local electronic medical registry. T2DM participants with a recent diagnosis were requested to have between 30 and 55 years, with < 3 years of disease duration. These criteria were chosen to create more homogenate clinical profiles of the three groups of patients with T2DM. The three clinical groups were selected since they are most prevalent in primary healthcare settings (18, 19). All participants with other types of diabetes (type 1, secondary, gestational, other) and those with medical history in the primary healthcare center for < 1 year were excluded. The participant selection sampling strategy was a convenient approach based on the availability and willingness of the participants who fulfilled the study criteria to participate.

### 2.3. Clinical practice guidelines

We evaluate the adherence to the different guidelines available and in force at the moment of realization of the study. Guidelines were classified into international, national, and local. The treatment recommendations for the three clinical groups of T2DM were compared among the guidelines. Only therapeutic recommendations that were similar among the different guidelines were considered to evaluate adherence and knowledge. Supplementary Table 1 shows the guidelines used in the evaluation of their adherence.

### 2.4. Definition of variables

During phase one, participant's inclusion visits, different sociodemographic and clinical variables were collected, such as age, sex, toxic habits, hypertension, hyperlipidemia, other clinically important diseases, concomitant treatment, laboratory parameters, BMI, and diabetes duration. For each group of participants (obese, frail, and recently diagnosed), antidiabetic treatment at the moment of inclusion was collected. Different pharmacologic groups of antidiabetic treatment were analyzed (metformin, sulfonylureas, glinides, thiazolidinedione-TZDs, dipeptidyl peptidase 4 (DPP-4) inhibitors, sodium-glucose co-transporter 2 (SGLT2) inhibitors, glucagon-like peptide-1 (GLP-1) analogs, fast-acting insulin, slow-acting insulin, intermedia acting insulin and mix action insulin). Based on this information, the variable “degree of adherence to therapeutic guidelines” was created using a scoring system. If the prescribed antidiabetic treatment was not recommended (contraindicated) by the guidelines for the specific participant group, a negative score (−1) was assigned. If the prescribed antidiabetic treatment was the one without specific recommendations or contraindications by the guidelines, a score of “0” was assigned. If the T2DM guidelines recommended the prescribed treatment, a score of “1” was assigned. In the case of multiple antidiabetic treatments, the scores were summarized. If the person had an overall negative score, it was classified as “inadequate adherence to the CPG.” If the overall scoring was 0 was classified as “average adherence to the CPG.” If the overall score was one or more, it was classified as “adequate adherence to the CPG.”

During phase two, we collected variables from professionals participating in the study, such as sex, if they have diabetes, professional experience, information related to the type of medical consultation, and indicators for the health care provided to participants with diabetes (number of participants with T2DM treated, T2DM visits and time per person). Moreover, the degree of knowledge related to CPG was also collected from professionals based on the specific questionnaire on recommendations from the therapeutic guidelines for the three types of T2DM participants. Three groups of “degree of CPG knowledge” were created based on the quartiles obtained from the correct answers to the questionnaire. Low CPG knowledge if the professional has an overall score of at least 9. Moderate CPG knowledge if the overall score was between 9 and 12. High CPG knowledge if the overall score was more than 12. Supplementary Table 2 shows the different items used to evaluate the degree of CPG knowledge.

### 2.5. Statistical analysis

The descriptive analyzes of phase one were carried out in the population of evaluable subjects by T2DM groups: subjects with obesity, frailty, and recently diagnosed, carrying out the comparative analyzes between the groups. The quantitative variables were described with measures of centralization and dispersion (mean, median, SD-standard deviation). Absolute and relative frequencies are described as qualitative variables. In the comparative analysis, depending on the characteristics of the variables and the number of groups being compared, parametric tests were used for those continuous variables that meet the application conditions (for example, *t*-test, ANOVA, etc.) and the non-parametric ones (for example, Chi-square, Fisher, Kruskal-Wallis, etc.) for ordinal, categorical variables or those that do not meet said parametric criteria. The *p*-values < 0.05 were considered statistically significant. Multivariate analysis was performed to determine related factors with a low level of knowledge using logistic regression, showing the Odds Ratio (OR) and its corresponding 95% confidence interval. In the model, professionals with moderate and high knowledge were merged into one variable to create a dichotomous depending variable. Missing data were not imputed and were left as missing. The data were analyzed using SPSS v22.0.

## 3. Results

In total, 262 primary care physicians were invited to participate; from those, 240 could be evaluated and included in the analysis. The participating physicians screened about 891 patients; from those, eight did not meet the study criteria and were excluded from the analysis. Supplementary Figure 1 shows the study flowchart.

### 3.1. Characteristics of the participants with diabetes

Participants with obesity and recently diagnosed with T2DM were mainly males (64.5% and 63.9%, respectively), while we observed more females (50.5%) in the frail group. Regarding the toxic habits, among the recently diagnosed participants, we observed more current smokers and alcohol consumers. The comorbidity profile was worst among the frail participants with T2DM for hypertension, CKD, and cardiovascular disease. At the same time, among the obese subjects, hyperlipidemia and mental illnesses were more prevalent than in the rest of the groups. Regarding laboratory parameters, no clinically significant differences were observed for HbA1c among the three groups. A poorer lipid profile was observed among the participants with a recent diagnosis of T2DM, and a poorer renal profile among the frail participants. Participants in the obese group mainly used lipid-lowering drugs. The remaining concomitant treatments (antihypertensive, antiplatelet, and anticoagulant drugs) were mostly used in the frail group. Regarding the antidiabetic drugs, metformin, DPP-4i, and slow-acting insulins were mainly used by frail people, while participants in the obese group mainly used SGLT-2i and GLP1-RA. We observed differences among the three groups for the number of medical visits related to diabetes in the last 12 months with physicians and nurses. The participants with obesity and T2DM had the highest, while participants with a recent diagnosis of T2DM had the lowest average number of visits with the professionals (4.3 visits ±3.9). The inadequate adherence to therapeutic guidelines was observed mainly among the participants in the obese and frail groups (3.4% and 3.5%, respectively). An average degree of adherence to the CPG was mainly observed among the participants in the frail group. In contrast, adequate adherence to the CPG was mostly observed among the newly diagnosed participants with T2DM. Table 1 summarizes the characteristics of the subjects included in the study.

**Table 1:** Characteristics of the participants.

	**Obese T2DM subjects *n* = 296**	**Frail T2DM subjects *n* = 289**	**Recently diagnosed T2DM subjects *n* = 296**	***p*-value**
**Sociodemographic and toxic habits**				
Age, mean (SD), years	63.0 (4.5)	79.8 (4.2)	49.0 (5.3)	<0.001^*^
Sex, *n* (%), male	191 (64.5)	143 (49.5)	189 (63.9)	<0.001^**^
Alcohol consumption^+^, *n* (%)	63 (21.3)	34 (11.8)	80 (27.0)	<0.001^**^
Current Smoking, *n* (%)	59 (19.9)	23 (8.0)	90 (30.4)	<0.001^**^
**Comorbidities**, ***n*** **(%)**				
Hypertension	239 (80.7)	245 (84.8)	133 (44.9)	<0.001^**^
Hyperlipidemia	252 (85.1)	221 (76.5)	189 (63.9)	<0.001^**^
Cardiovascular disease	3 (1.0)	20 (6.9)	2 (0.7)	<0.001^**^
Chronic kidney disease	38 (12.8)	81 (28.0)	19 (6.4)	<0.001^**^
Relevant mental illness	38 (12.8)	51 (17.6)	39 (13.2)	0.184^**^
**Clinical variables**				
Diabetes duration (years)	11.2 (6.0)	15.1 (8.0)	1.8 (0.9)	<0.001^*^
BMI, mean, (SD)	33.4 (2.9)	29.7 (4.5)	29.8 (5.0)	<0.001^*^
**Laboratory parameters**				
HbA1c (%), mean, (SD)	7.4 (1.1)	7.4 (1.3)	7.3 (1.3)	0.037^*^
Triglycerides (mg/dL),mean, (SD)	177.7 (78.0)	159.1 (68.0)	177.5 (79.6)	0.002^*^
Cholesterol total (mg/dL),mean, (SD)	199.5 (44.4)	186.5 (45.3)	201.9 (43.1)	<0.001^*^
Cholesterol LDL (mg/dL),mean, (SD)	116.1 (40.2)	106.1 (36.8)	121.1 (36.2)	<0.001^*^
Glomerular filtration (mL/min/1.73 m^2^), (SD)	76.8 (18.6)	68.2 (19.6)	84.0 (18.9)	<0.001^*^
Albumin/creatinine ratio (mg/dL),mean, (SD)	51.8 (184.4)	80.2 (305.1)	46.7 (223.8)	0.003^*^
**Concomitant treatments**, ***n*** **(%)**				
Antihypertensive drugs	237 (80.1)	244 (84.4)	132 (44.6)	<0.001^**^
Lipid-lowering drugs	250 (84.5)	220 (76.1)	177 (59.8)	<0.001^**^
Antiplatelet drugs	102 (34.5)	131 (45.3)	40 (13.5)	<0.001^**^
Anticoagulant drugs	14 (4.7)	36 (12.5)	10 (3.4)	<0.001^**^
**Glucose lowering drugs**, ***n*** **(%)**				
Metformin	217 (73.3)	230 (79.6)	228 (77.0)	0.196^**^
SU	18 (6.1)	36 (12.5)	17 (5.7)	0.004^**^
Glinides	11 (3.7)	34 (11.8)	9 (3.0)	<0.001^**^
TZDs	1 (0.3)	4 (1.4)	1(0.3)	0.296^***^
DPP-4i	185 (62.5)	224 (77.5)	139 (47.0)	<0.001^**^
SGLT-2i	95 (32.1)	68 (23.5)	76 (25.7)	0.052^**^
GLP1-RA	35 (11.8)	19 (6.6)	16 (5.4)	0.009^**^
Insulin fast acting	5 (1.7)	7 (2.4)	4 (1.4)	0.612^**^
Insulin slow acting	35 (11.8)	61 (21.1)	22 (7.4)	<0.001^**^
Insulin intermedia acting	0 (0.0)	6 (2.1)	0 (0.0)	0.001^***^
Insulin mix action	2 (0.7)	7 (2.4)	1 (0.3)	0.066^**^
**Number of visits related to diabetes in the last 12 months, mean, (SD)**				
Primary care physician	4.3 (3.9)	4.2 (3.3)	3.3 (2.6)	<0.001^*^
Primary care nurse	4.4 (5.7)	4.3 (4.0)	3.4 (3.4)	<0.001^*^
Adherence to therapeutic guidelines, *n* (%)				<0.001^*^
Inadequate	10 (3.4)	10 (3.5)	0 (0.0)	
Average	45 (15.2)	63 (21.8)	25 (8.4)	
Adequate	241 (81.4)	216 (74.7)	271 (91.6)	

BMI, body mass index; HbA1c, glycosylated hemoglobin; ^+^Moderate and high-risk alcohol consumption; DPP-4i, dipeptidyl peptidase 4 (DPP-4) inhibitors; SGLT-2i, sodium-glucose co-transporter 2 (SGLT2) inhibitors; GLP1-RA, glucagon-like peptide-1 (GLP-1) analogs; TZDs, thiazolidinediones; T2DM, type 2 diabetes mellitus; SU, sulphonylureas; SD, standard deviation; NIAID, non-insulin antidiabetic drugs.

Statistical tests used: (^*^) Kruskal-Wallis, (^**^) Chi-square, (^***^) Fisher.

### 3.2. Characteristics of the professionals

Most of the professionals (primary care physicians) included in the analysis were males. About 8.3% reported having T2DM. We observed that physicians included in our study had, on average, 26.4 years of professional experience, 16.7% were members of some diabetes working group, and 74.2% had some educational course related to diabetes in the last 12 months. The majority (69.2%) worked in urban primary health care centers; on average, 225.3 patients with T2DM in their consultation, and the average time for consultation per person with T2DM was 11.3 min. Stratified for the degree of knowledge, the majority, 137 (57%) of the primary care physicians, had a moderate degree of CPG knowledge based on the answers to the study questionnaire. Supplementary Table 2 shows the descriptive analysis of the answers to the study questionnaire used to estimate the degree of CPG knowledge. We did not observe statistically significant differences among the three degrees of CPG knowledge. However, the low-degree group had perceptually more males, on average 27.4 years of professional experience, and with more experience in participation in the clinical trial for diabetes and other metabolic/cardiovascular/renal diseases in the last 12 months. The professionals from this group were the majority from semi-urban primary care centers with the lowest number of patients in the quota and subjects with diabetes. Table 2 summarizes the characteristics of primary care physicians.

**Table 2:** Characteristics of the professionals and their degree of CPG knowledge.

	**Total**	**Degree of CPG knowledge**			
	**Physicians** ***n*** = **240**	**Low (*****n*** = **61)**	**Moderate (*****n*** = **137)**	**High (*****n*** = **42)**	***p*** **-value**
Sex, *n* (%), male	167 (69.6)	46 (75.4)	94 (68.6)	27 (64.3)	0.450^**^
**Comorbidities**, ***n*** **(%)**					
Type 2 diabetes presence	20 (8.3)	4 (6.6)	15 (10.9)	1 (2.4)	0.207^***^
**Experience**					
Years of professional experience, mean (SD)	26.4 (9.2)	27.4 (9.0)	26.6 (9.3)	24.5 (8.8)	0.167^*^
Years in the last job mean (SD)	14.6 (9.9)	14.5 (9.9)	14.8(10.0)	14.0 (9.9)	0.923^*^
Member of a diabetes working group	40 (16.7)	6 (9.8)	27 (19.7)	7 (16.7)	0.227^**^
Courses/professional education related to diabetes management in the last 12 months, *n* (%)	178 (74.2)	40 (65.6)	107 (78.1)	31 (73.8)	0.177^**^
Participation in clinical trials for diabetes and other metabolic/cardiovascular/renal diseases in the last 12 months, *n* (%)	59 (24.6)	18 (29.5)	32 (23.4)	9 (21.4)	0.567^**^
**Place of work**					
Urban	166 (69.2)	39 (63.9)	97 (70.8)	30 (71.4)	0.899^**^
Semi-urban	44 (18.3)	13 (21.3)	24 (17.5)	7 (16.7)	
Rural	30 (12.5)	9 (14.8)	16 (11.7)	5 (11.9)	
Patients in the quota, mean, (SD)	1644.4 (701.4)	1460.4 (587.9)	1751.8 (805.0)	1565.6 (342.5)	0.244^*^
Participants with diabetes in the quota mean (SD)	225.3 (180.7)	191.1 (133.2)	246.1 (211.8)	208.1 (110.9)	0.382^*^
Participants with T2DM who visit weekly mean (SD)	27.4 (39.3)	28.2 (43.4)	29.5 (42.6)	19.4 (11.6)	0.224^*^
Duration of T2DM consultation per person (min) mean, (SD)	11.3 (4.5)	11.2 (4.4)	11.6 (4.9)	10.5 (3.1)	0.603^*^

CPG, clinical practice guidelines; SD, standard deviation.

Statistical tests used: (^*^) Kruskal-Wallis, (^**^) Chi-square, (^***^) Fisher.

### 3.3. The degree of adherence to the CPGs in the three clinical situations

Stratifying the degree of adherence to the CPG and the professionals' CPG knowledge, we observed that inadequate adherence to the guidelines was mainly observed only among the obese and frail participants with diabetes from medical doctors with a low degree of CPG knowledge according to the study questionnaire. The average adherence to the CPG was mainly present among frail participants (61.0%) from professionals with moderate knowledge. Adequate adherence to the guidelines was mainly achieved among the recently diagnosed participants with T2DM (58.5%), especially among the patients from professionals with a moderate degree of CPG knowledge.

Regarding the patient's characteristics and the degree of adherence to the guidelines, the participants with inadequate adherence to the guidelines were generally older, with higher BMI, poorer HbA1c control, and fewer visits with primary care physicians. The patients with average adherence to the guidelines had the lowest average BMI and HbA1c and a higher average number of visits with their medical doctor. The lowest average HbA1c was observed among the recently diagnosed T2DM subjects with average adherence to the CPG. We observed a higher percentage with proper HbA1c control (HbA1c < 7%) among the recently diagnosed T2DM individuals and professionals with adequate CPG knowledge. The highest average number of visits with the medical doctor was observed among the obese participants with T2DM with average adherence to the CPG. Table 3 summarizes the results related to adherence with the CPGs in the three clinical situations.

**Table 3:** Degree of compliance to the guidelines stratified by professional's different degrees of knowledge and patients characteristics.

		**Degree of adherence to the CPG**											
		**Inadequate**				**Average**				**Adequate**			
		**Overall *n* = 20**	**Obese *n* = 10**	**Frail *n* = 10**	**Recently diagnosed *n* = 0**	**Overall *n* = 120**	**Obese *n* = 39**	**Frail *n* = 59**	**Recently diagnosed *n* = 22**	**Overall *n* = 685**	**Obese *n* = 227**	**Frail *n* = 205**	**Recently diagnosed *n* = 253**
Patients from professionals with different degrees of knowledge	Low (*n* = 209)	10 (50.0)	5 (50.0)	5 (50.0)	–	39 (32.5)	18 (46.2)	12 (20.3)	9 (40.9)	160 (23.4)	47 (20.7)	52 (25.4)	61 (24.1)
	Moderate (*n* = 478)	8 (40.0)	4 (40.0)	4 (40.0)	–	63 (52.5)	16 (41.0)	36 (61.0)	11 (50.0)	407 (59.4)	140 (61.7)	119 (58.0)	148 (58.5)
	High (*n* = 138)	2 (10.0)	1 (10.0)	1 (10.0)	–	18 (15.0)	5 (12.8)	11 (18.6)	2 (9.1)	118 (17.2)	40 (17.6)	34 (16.6)	44 (17.4)
Patient characteristics	Age, mean, (SD)	71.4 (7.0)	65.0 (2.9)	77.8 (2.1)	–	68.3 (12.5)	62.9 (4.4)	79.6 (4.5)	49.4 (3.6)	62.8 (13.5)	62.9 (4.6)	79.9 (4.2)	49.0 (5.4)
	Sex (male), *n* (%)	14 (70.0)	6 (60.0)	8 (80.0)	–	69 (51.9)	28 (62.2)	26 (41.3)	15 (60.0)	440 (60.4)	157 (65.1)	109 (50.5)	174 (64.2)
	Alcohol consumption, *n* (%)	4 (20.0)	2 (20.0)	2 (20.0)	–	23 (17.3)	9 (20.0)	7 (11.1)	7 (28.0)–	150 (20.6)	52 (21.6)	25 (11.6)	73 (26.9)
	Tabaco consumption, *n* (%)	5 (25.0)	3 (30.0)	2 (20.0)	–	20 (15.0)	7 (15.6)	3 (4.8)	10 (40.0)	147 (20.2)	49 (20.3)	18 (8.3)	80 (29.5)
	BMI mean, (SD)	31.8 (5.0)	35.5 (3.1)	28.2 (3.6)	–	30.5 (4.2)	32.3 (2.4)	30.3 (4.8)	27.9 (3.5)	31.0 (4.6)	33.5 (2.9)	29.6 (4.4)	30.0 (5.0)
	HbA1c mean, (SD)	7.5 (1.0)	7.8 (0.8)	7.2 (1.1)	–	7.3 (1.1)	7.2 (1.0)	7.5 (1.2)	7.0 (1.1)	7.4 (1.3)	7.4 (1.1)	7.4 (1.3)	7.3 (1.3)
	HbA1c < 7%, *n* (%)	6 (30.0)	1 (10.0)	5 (50.0)	–	50 (37.6)	17 (37.8)	22 (34.9)	11 (44.0)	282 (38.7)	84 (34.9)	76 (35.2)	122 (45.0)
	HbA1c < 8%, *n* (%)	13 (65.0)	6 (60.0)	7 (70.0)	–	102 (76.7)	34 (75.6)	48 (76.2)	20 (80.0)	552 (75.8)	182 (75.5)	158 (73.1)	212 (78.2)
	Glomerular filtration mean, (SD)	72.2 (14.7)	76.1 (16.5)	68.4 (12.4)	–	70.7 (20.0)	73.8 (22.5)	69.0 (15.6)	69.6 (24.9)	77.6 (20.1)	77.4 (17.8)	68.0 (20.9)	85.3 (17.8)
	CAC mean, (SD)	16.5 (16.0)	12.8 (17.0)	20.2 (14.9)	–	58.7 (264.6)	23.7 (33.0)	94.9 (380.7)	30.7 (45.9)	60.7 (241.9)	58.7 (203.3)	78.7 (287.5)	48.2 (233.5)
	Number of visits with a physician mean, (SD)	3.4 (1.7)	3.7 (2.1)	3.0 (1.3)	–	4.5 (4.0)	5.9 (5.6)	3.7 (2.5)	4.0 (2.8)	3.8 (3.3)	4.0 (3.5)	4.4 (3.6)	3.2 (2.6)

BMI, body mass index; CPG, clinical practice guidelines; HbA1c, glycosylated hemoglobin; ^+^Moderate and high-risk alcohol consumption; SD, standard deviation.

### 3.4. Factors related to the low level of CPG knowledge among the professionals

**Figure 1:**
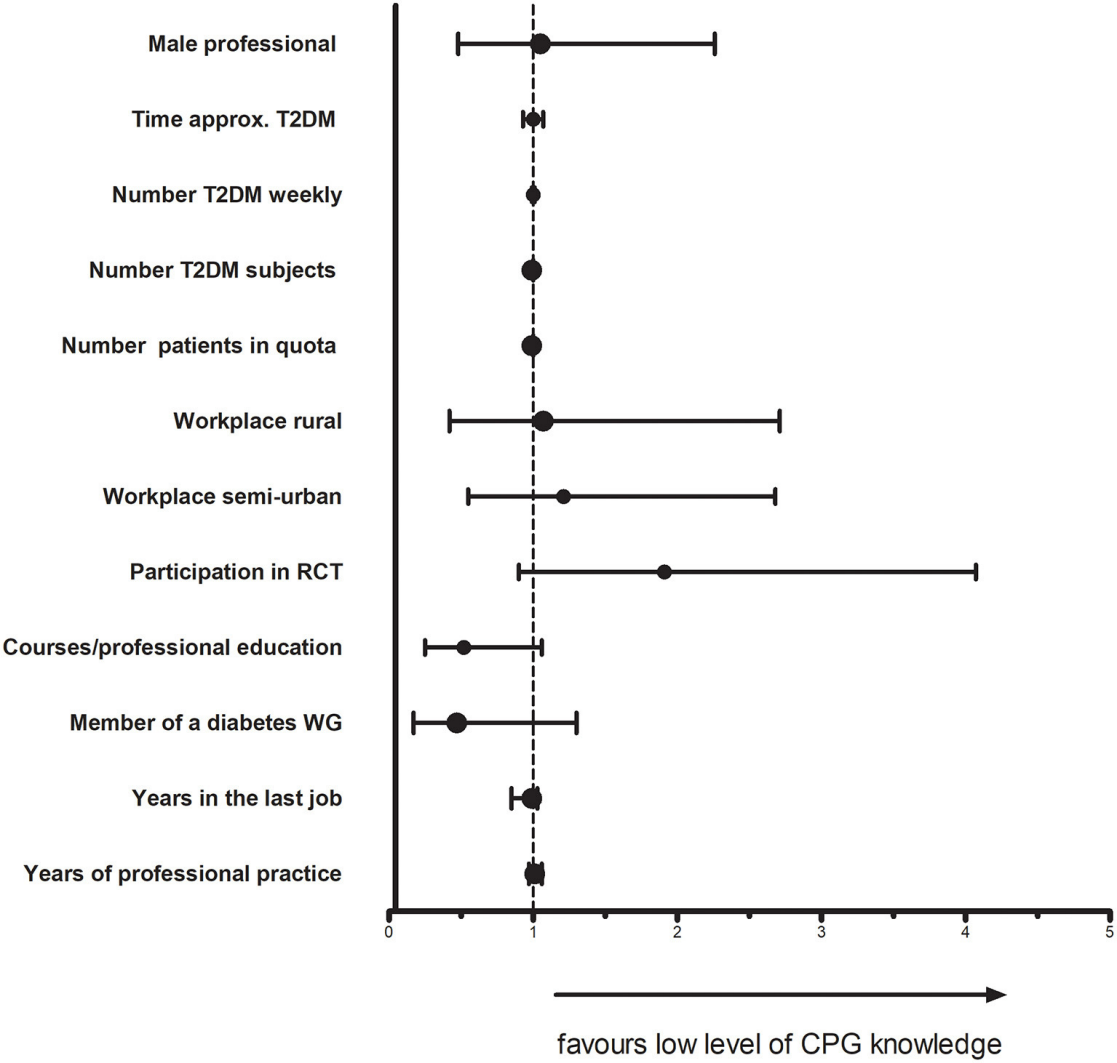
Factors related to the low level of CPG knowledge. CPG, clinical practice guidelines; RCT, randomized clinical tiral; T2DM, type 2 diabetes melitus; WG, working group.

In the multivariable analysis, considering different potential factors for a low level of CPG knowledge among the professionals, we did not observe any statistically significant odds ratios. Figure 1 and Supplementary Table 3 show the results of this analysis.

## 4. Discussion

The results of our cross-sectional analysis on the degree of adherence with the therapeutic recommendations of the CPG among obese, frail, and recently diagnosed T2DM subjects in primary care centers in Spain show adequate adherence for most participants. This percentage was highest among the recently diagnosed T2DM subjects.

Similar studies evaluating adherence to the CPG were done in other countries but with different methodologies and objectives, making comparing difficult with our study. For example, cohort study from Luxemburg with 21,068 T2DM subjects for the period between 2000 and 2006, the authors evaluate the adherence of physicians and patients to annual follow-up recommendations from international guidelines for T2DM subjects (20). The authors reported that 90% of the patients consulted more than four times their treating physician. In our study, the average number of visits annually with the treating physician was different for three groups of participants, the highest number of visits among subjects with obesity (4.3 visits) and the lowest among the subjects with recently diagnosed T2DM (3.3 visits). Notably, the number of visits was higher among the obese than the frail persons, whose clinical conditions are more changeable due to their clinical complexity. A study from rural northern Alberta, Canada had, aim to evaluate adherence with the local clinical guidelines for clinical indicators and therapeutic targets on 368 patients with T2DM. The authors reported that, on average, the clinical indicators were near the recommended clinical practice guideline targets (49.7% with HbA1C < 7%) (21). Another similar study from Swiss evaluated adherence with the local clinical guidelines target criteria for good disease management of diabetes in 604 patients. The authors reported that 44% of the patients achieved the therapeutic target [HbA1c < 7% (53 mmol/mol)]. At the same time, this percentage was higher (77%) for the target [HbA1c < 8% (64 mmol/mol)]. Compared with our results, 38.7% of the participants with adequate adherence with CPG had achieved the target of HbA1c < 7%, and 75.8% achieved the target of HbA1c < 8%. On the other hand, in Greece, recently, one study was published evaluating the level of adoption and adherence to local T2DM therapeutic guidelines among 226 Greek physicians (22). The authors reported that among the investigated physicians, there was a high level of adaptation to the guidelines (92.2%). However, the authors reported a low adherence (26.1%) to CPG. In our study, most physicians (57.0%) had average knowledge of therapeutic guidelines. In our multivariable model, we did not observe any statistically significant odds ratios for different characteristics of the professionals, consultation size, and low levels of CPG knowledge. One study in the U.K. evaluated the medical consultation size with practice performance and quality of care. The authors reported similar quality of care between the practices with larger numbers of patients and those with fewer patients per doctor (23). A recently published cross-sectional study assessing factors involved in adherence to CPGs on T2DM diabetes among the 98 endocrinologists working in public hospitals from Spain reported that non-adherence to CPG was a multifactorial problem related with the existence of multiple CPGs, the therapeutic inertia, the lack of time, and the complexity of diabetes (24).

Antidiabetic drugs should be selected for most of the current CPG, considering the individual patient's clinical characteristics and glycemic objectives (25). In the case of obesity and T2DM, the Standards of Medical Care in Diabetes from the American Diabetes Association for 2022, for patients where the objective is weight reduction, recommends the use of pharmacological agents with evidence of weight loss, such as metformin, α-glucosidase inhibitors, SGLT-2i, GLP1-RA, and amylin mimetics (26). Compared with our results, most participants with T2DM and obesity were using metformin, and surprisingly in second place (62.5%) were using weight-neutral drugs such as DPP-4i. About 11.8% and 6.1% of the participants with obesity were using weight-increasing drugs (insulins or sulphonylureas, respectively).

Concerning older adults and frailty, the international guidelines recommend metformin as first-line treatment if no contraindications exist (advanced renal insufficiency, impaired hepatic function) or gastrointestinal side effects which could reduce appetite or provoke vitamin B12 deficiency (27). In our study, most of the participants in the frail group were on metformin, while in second place by the use were the DPP-4inhibitors. In general, if no contraindication exit, DPP-4 inhibitors are also recommended due to the few side effects and minimal risk of hypoglycemia. Slow-acting insulins were mainly prescribed to these subjects. Once-daily slow-acting (basal) insulin has a good safety profile and is often used in older adults (28). A recently published review on diabetes and frailty suggests that as patients with diabetes get older, it is recommended to simplification, switch, or de-escalate the antidiabetic treatment depending on the frailty or HbA1Cc levels (22). In general, avoiding treatments that could induce hypoglycemia, such as sulphonylureas and fast-acting insulins, is suggested. Among our frail participants, 12.5% used some sulphonylureas, and 2.4% used some fast-acting insulins.

Regarding patients with a recent diagnosis of T2DM, most guidelines recommend using metformin as a first-line treatment when glycemic objectives are not reached with no pharmacological measures (dietary changes or physical exercises). In our study, most of the participants in this group were using metformin. In the recent therapeutic local guidelines from the RedGDPS foundation in Spain, in the case of young or recently diagnosed T2DM participants, the recommendation for the therapeutic objective is HbA1c < 6.5% in monotherapy or non-pharmacological treatment, avoiding drugs with a risk of hypoglycemia (29). Our study observed that the proportion of subjects on some antidiabetic treatment that could cause hypoglycemia, such as sulphonylureas or insulins, was lower among the three groups. Proper therapeutic management in these recently diagnosed patients is important, especially in preventing possible complications or achieving correct therapeutic adherence.

We have to acknowledge some limitations in our study. Firstly, this was a cross-sectional observational study, where participants were selected due to their clinical profile, so inherent selection biases are possible. Secondly, the data collection was retrospective from the participant's medical records, so possible missing some variables possible, such as laboratory parameters or clinical variables related to physical examination, etc. On the other hand, the questionaries' used to evaluate a professional's degree of knowledge are not standardized or validated but just related to the recommendations available in current clinical guidelines. Moreover, those questionnaires were auto-administrated, so there is a possibility that person who was answering the questions could look for the answers in the clinical guidelines even though that time for answering was limited. Thirdly, additional inclusion criteria are just approximations for the definition of the three groups of T2DM patients, no specific test or functional probes were performed to identify/quantify the frailty (Barthel index or Lawton scale). Fourthly, the adherence to antidiabetic drugs was not evaluated in this study. Fifthly the professionals were not selected randomly but simply invited to participate in the study; therefore, there is a possibility of selection bias or that only highly motivated primary healthcare professionals took part in this study. Finally, the sample size in this paper is small, and the population is divided into three groups, which further weakens the statistical efficacy. The strengths of our study are the specific study design focused on professionals and patients, the number of participants, and the settings of realization. Primary care centers are the healthcare gate for participants with diabetes, they represent the clinical reality, and the results of this analysis have high external validity.

In conclusion, our cross-sectional analysis shows adequate adherence to the clinical guidelines by the primary care physicians for most participants with obesity, frailty, or newly diagnosed with T2DM. Specifically designed interventions are needed for professionals and participants with diabetes to improve the control and complications of type 2 diabetes in primary health care settings.

## Data availability statement

The datasets presented in this article are not readily available due to the legal limitations, the dataset of this study is a property of RedGDPS Foundation. Requests to access the datasets should be directed to JF-N, josep.franch@gmail.com.

## Ethics statement

The study involves human participants, and it was reviewed and approved by IDIAP Jordi Gol Ethics Committee (protocol approval number 20/098-P, on 02/07/2020). The patients/participants provided their written informed consent to participate in this study.

## Author contributions

Conceptualization, methodology, and writing: BV and JF-N. Original draft preparation: BV. Review and editing: BV, MM-C, LR, JB-d, BF-C, and JF-N. Supervision and funding acquisition: JF-N. All authors have read and agreed to the published version of the manuscript.
