# Supplementary material for: Adherence to the therapeutic guidelines recommendations among the people with type 2 diabetes mellitus and obesity, frailty, or recent diagnosis, attended in primary health care centers in Spain: A cross-sectional study
Source: Front Med (Lausanne). 2023 Mar 24;10:1138956. doi: 10.3389/fmed.2023.1138956 (PMC10080116; doi:10.3389/fmed.2023.1138956)
Supplement: Supplementary file 1 [file Table_1.docx]

**Supplementary Materials**

Adherence to the therapeutic guidelines recommendations among the people with type 2 diabetes mellitus and obesity, frailty, or recent diagnosis, attended in primary health care centers in Spain: a cross-sectional study

**Bogdan Vlacho , Manel Mata-Cases , Berta Fernandez-Camins , Laura Romera Liébana, Joan Barrot-de la Puente , Josep Franch-Nadal***

|  | **Page** |
| --- | --- |
| **Supplementary Table 1.** International and National guidelines are considered for analysis of adherence | **2** |
| **Supplementary figure 1.** Study flowchart | **4** |
| **Supplementary Table 2.** Questionnaire items descriptive for each item | **5** |
| **Supplement Table 3.** Multivariate model for a low level of knowledge | **6** |

**Supplementary Table 1.** International and National guidelines are considered for analysis of adherence

| **International guidelines** |
| --- |
| - ADA/EASD consensus report, see Davies MJ, D’Alessio DA, Fradkin J et al. (2018). Management of hyperglycaemia in type 2 diabetes. A consensus report by the American Diabetes Association (ADA) and the European Association for the Study of Diabetes (EASD). Diabetology , 2018; 61(12), 2461–2498. The complete guideline is available at: care.diabetesjournals.org/content/41/12/2669. - NICE Guideline 28, see: National Institute for Health and Care Excellence. Type 2 diabetes in adults: management. NICE Guideline 28. NICE, 2017. The complete guideline is available at: [www.nice](http://www.nice).org.uk/guidance/ng28 |
| **National guidelines** |
| - Recommendations of the redGDPS , see: Alemán Sánchez JJ, Artola Menéndez S, Ávila Lachica L, Barrot de la Puente Joan et al. Type 2 Diabetes Guide for Clinicians: Recommendations from the GDPS Network . The complete guide is available at: <https://www>.redgdps.org/guia-de-diabetes-tipo-2-para-clinicos/ - Recommendations of the Spanish Society of Diabetes (SED ), see: Gomez –Peralta F, Escalada San Martín FJ, Menéndez Torre E, Mata Cases M et al. Recommendations of the Spanish Society of Diabetes (SED) for the pharmacological treatment of hyperglycemia in type 2 diabetes: 2018 update. Endocrinol Diabetes y Nutr [Internet]. 2018 Dec ;65 (10):611–24. Available at: <https://linkinghub>.elsevier.com/retrieve/pii/S2530016418301940 - Recommendations in the pharmacological treatment of DM2 of the Diabetes, Obesity and Nutrition Group of the SEMI. Half [Internet]. 2019;211–2. Available from : <https://www>.fesemi.org/sites/default/files/documentos/publicaciones/algoritmo-dm-semi-2019.pdf |
| **Local guidelines** |
| - *Andalucía: Servicio Andaluz de Salud*   *PROCESO ASISTENCIAL INTEGRADO. DIABETES MELLITUS*  *Edita: Junta de Andalucía. Consejería de Salud.* [*https://www.juntadeandalucia.es/export/drupaljda/diabetes_mellitus_2018_18_06_2018.pdf*](https://www.juntadeandalucia.es/export/drupaljda/diabetes_mellitus_2018_18_06_2018.pdf)   - *Aragón : Servicio Aragonés de Salud*   *Atención Integral a la Diabetes en Aragón*  [*https://www.aragon.es/documents/20127/674325/Programa%20Atencion%20Integral%20Diabetes%20Mellitus%20Aragon.pdf/726e9d57-837c-9a6c-f68e-9038cb2b0733*](https://www.aragon.es/documents/20127/674325/Programa%20Atencion%20Integral%20Diabetes%20Mellitus%20Aragon.pdf/726e9d57-837c-9a6c-f68e-9038cb2b0733)   - *Asturias : Servicio de Salud del Principado de Asturias*   *Actualización del PCAI Diabetes – Astursalud*  [*https://seapaonline.org/UserFiles/File/Ayuda%20en%20consulta/pcais/diabetes.pdf*](https://seapaonline.org/UserFiles/File/Ayuda%20en%20consulta/pcais/diabetes.pdf)   - *Baleares : Servei de Salut de les Illes Balears*   [*https://www.ibsalut.es/apmallorca/attachments/article/1006/estrategia-de-diabetis-ib-ca.pdf*](https://www.ibsalut.es/apmallorca/attachments/article/1006/estrategia-de-diabetis-ib-ca.pdf)   - *Canarias : Servicio Canario de la Salud*   [*https://www3.gobiernodecanarias.org/sanidad/scs///content/a539bd6a-cf08-11e4-b8de-159dab37263e/07_Abordaje_Diabetes.pdf*](https://www3.gobiernodecanarias.org/sanidad/scs///content/a539bd6a-cf08-11e4-b8de-159dab37263e/07_Abordaje_Diabetes.pdf)   - *Cantabria :*   [*http://saludcantabria.es/index.php?page=profesional*](http://saludcantabria.es/index.php?page=profesional)   - *Castilla-La Mancha : Servicio de Salud de Castilla-La Mancha*   [*http://pagina.jccm.es/sanidad/salud/plandm.pdf*](http://pagina.jccm.es/sanidad/salud/plandm.pdf)   - *Castilla y León : Sanidad de Castilla y León*   [*https://www.saludcastillayleon.es/profesionales/es/cardiovascular/documentacion/guias/guia-practica-clinica-diabetes-mellitus-tipo-2*](https://www.saludcastillayleon.es/profesionales/es/cardiovascular/documentacion/guias/guia-practica-clinica-diabetes-mellitus-tipo-2)   - *Cataluña : Servei Català de la Salut*   [*https://catsalut.gencat.cat/web/.content/minisite/catsalut/proveidors_professionals/medicaments_farmacia/harmonitzacio/pautes/diabetis-mellitus-tipus2/pauta-harmonitzacio-diabetis-mellitus-tipus2.pdf*](https://catsalut.gencat.cat/web/.content/minisite/catsalut/proveidors_professionals/medicaments_farmacia/harmonitzacio/pautes/diabetis-mellitus-tipus2/pauta-harmonitzacio-diabetis-mellitus-tipus2.pdf)   - *Comunidad Valenciana : Conselleria de Sanitat Universal i Salut Pública* *Generalitat Valenciana*   [*http://publicaciones.san.gva.es/publicaciones/documentos/V.5221-2008.pdf*](http://publicaciones.san.gva.es/publicaciones/documentos/V.5221-2008.pdf)   - *Extremadura : Servicio Extremeño de Salud (SES)*   [*https://saludextremadura.ses.es/filescms/web/uploaded_files/CustomContentResources/PlanIntegralDiabetes.pdf*](https://saludextremadura.ses.es/filescms/web/uploaded_files/CustomContentResources/PlanIntegralDiabetes.pdf)   - *Galicia : Servicio Gallego de Salud*   [*https://www.sergas.es/Asistencia-sanitaria/Dabetes-mellitus-tipo-2?idioma=es*](https://www.sergas.es/Asistencia-sanitaria/Dabetes-mellitus-tipo-2?idioma=es)   - *Madrid:* *Servicio Madrileño de Salud*   [*https://www.comunidad.madrid/servicios/salud/diabetes*](https://www.comunidad.madrid/servicios/salud/diabetes)   - *Murcia:* *Servicio Murciano de Salud*   [*https://www.murciasalud.es/publicaciones.php?op=mostrar&tipo=materias&id=1&anno=&puede_editar=&pagina=15*](https://www.murciasalud.es/publicaciones.php?op=mostrar&tipo=materias&id=1&anno=&puede_editar=&pagina=15)   - *Navarra : Servicio Navarro de Salud*   [*https://www.navarra.es/home_es/Temas/Portal+de+la+Salud/Ciudadania/Mi+enfermedad/Diabetes/*](https://www.navarra.es/home_es/Temas/Portal+de+la+Salud/Ciudadania/Mi+enfermedad/Diabetes/)   - *País Vasco : Departamento de Salud-* *Osakidetza*   [*https://www.osakidetza.euskadi.eus/contenidos/informacion/osteba_publicaciones/es_osteba/adjuntos/e_06_06_Diabetes_tipo_2a%20completa.pdf*](https://www.osakidetza.euskadi.eus/contenidos/informacion/osteba_publicaciones/es_osteba/adjuntos/e_06_06_Diabetes_tipo_2a%20completa.pdf)   - *La Rioja :* *Rioja Salud*   [*https://www.riojasalud.es/ciudadanos/catalogo-multimedia/endocrinologia/que-es-la-diabetes*](https://www.riojasalud.es/ciudadanos/catalogo-multimedia/endocrinologia/que-es-la-diabetes) |

**Supplementary figure 1.** Study flowchart

**
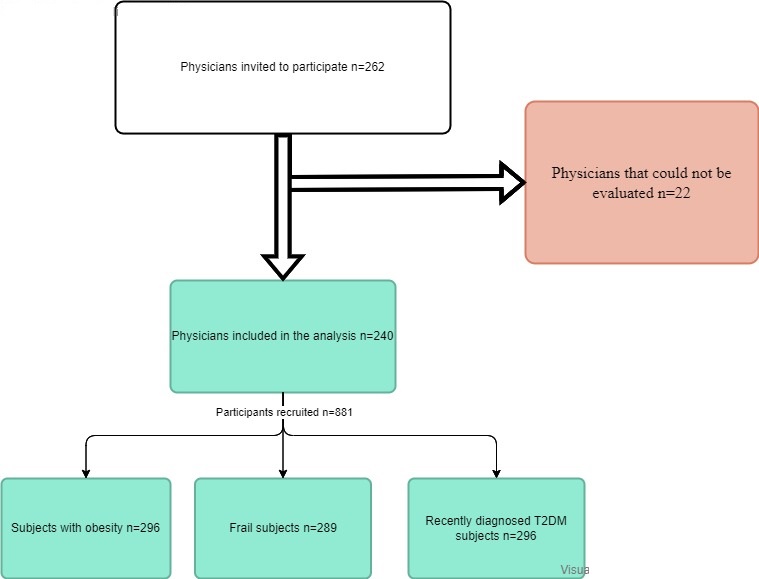
**

**Supplementary Table 2.** Questionnaire items descriptive for each item

| ITEMS | Possible answers | Number answers N=240 | (%) |
| --- | --- | --- | --- |
| *Antidiabetic drugs associated with clinical evidence of weight loss are: | Sulfonylureas | 11 | 4.6 |
|  | SGLT2I | 210 | 87.5 |
|  | Pioglitazone | 20 | 8.3 |
|  | arGLP1 | 199 | 82.9 |
|  | Insulins | 10 | 4.2 |
| In case of obesity (BMI>30 kg/m2) and poor glycemic control after therapeutic failure with metformin, the antidiabetic treatment intensification should be with: | Glibenclamide | 4 | 1.7 |
|  | Repaglinide | 9 | 3.8 |
|  | Insulins | 5 | 2.1 |
|  | arGLP1 | 222 | 92.5 |
| Treatment with metformin could be intensified in the case of an obese patient with poor glycemic control with a drug from the iSGLT-2 group only if: | IMC >45 kg/m^2^ | 27 | 11.3 |
|  | FGe < 30 ml/min/1,73m^2^ | 11 | 4.6 |
|  | FGe >60 ml/min/1,73m^2^ | 202 | 84.2 |
| In frail patients, the most recommended antidiabetic drug due to the safety evidence is: | Sulphonylureas | 6 | 2.5 |
|  | DPP-4i | 218 | 90.8 |
|  | SGLT-2i | 8 | 3.3 |
|  | arGLP1 | 3 | 1.3 |
|  | Insulins | 5 | 2.1 |
| *Which of these monotherapy antidiabetic drugs have no evidence of producing hypoglycemia? | Metformin | 137 | 57.1 |
|  | Sulphonylureas | - | - |
|  | DPP-4i | 186 | 77.5 |
|  | SGLT-2i | 109 | 45.4 |
| *In a patient with a glomerular filtration rate <30 ml/min, which of the following DPP4i could be prescribed? | Sitagliptin | 105 | 43.8 |
|  | Vildagliptin | 42 | 17.5 |
|  | Linagliptin | 171 | 71.3 |
|  | Alogliptin | 35 | 14.6 |
| In patients with a long evolution of T2DM (> 10 years), a history of severe hypoglycemia, microvascular and/or macrovascular complications, comorbidities, reduced life expectancy and/or biopsychosocial problems, the control objectives would be: | HbA1C<6.5% | 35 | 14.6 |
|  | HbA1C between 7 and 8.5% | 163 | 67.9 |
|  | HbA1C<10% | 29 | 12.1 |
|  | HbA1c control is not important | 13 | 5.4 |
| *If HbA1c>9% is detected at the onset of DM2, it is recommended | Maintain diet and exercise for only three months and reassess the introduction of drugs | 36 | 15.0 |
|  | Metformin monotherapy | 59 | 24.6 |
|  | Start combination therapy with two oral antidiabetics | 124 | 51.7 |
|  | A transitory insulinization | 64 | 26.7 |
| In participants with T2DM who have a history of CVD, it is recommended to add to treatment metformin, drugs that have been shown to clinically reduce cardiovascular events: | Empagliflozin , canagliflozin, liraglutide, and semaglutide | 228 | 95.0 |
|  | Glimepiride and Gliclazide retard | 2 | 0.8 |
|  | Insulin | 3 | 1.3 |
|  | Pioglitazone | 7 | 2.9 |
| *In adults with T2DM, do not offer or continue pioglitazone if they have any of the following symptoms: | Heart failure or a history of heart failure | 198 | 82.5 |
|  | Liver impairment | 119 | 49.6 |
|  | Insulin resistance | 17 | 7.1 |
|  | Current or history of bladder cancer | 119 | 49.6 |

BMI: Body mass index; CVD: Cardiovascular disease; HbA1c: glycosylated haemoglobin;; DPP-4i: Dipeptidyl peptidase 4 (DPP-4) inhibitors; SGLT-2i: Sodium-glucose co-transporter 2 (SGLT2) inhibitors; GLP1-RA: Glucagon-like peptide-1 (GLP-1) analogues; TZDs: Thiazolidinediones; T2DM: type 2 diabetes mellitus; SU: sulphonylureas;

*multiple answers were correct;

**Supplement Table 3.** Multivariate model for a low level of CPG knowledge

| **Independent variables** | **low level of CPG knowledge** | | | | | |
| --- | --- | --- | --- | --- | --- | --- |
|  | **Odds Ratio** | **CI 95% (OR)** | | **B** | **Standard Error** | **p-value** |
|  |  | **Limit inferior** | **Limit superior** |  |  |  |
| Years of professional practice | 1.02 | 0.97 | 1.06 | 0.02 | 0.02 | 0.47 |
| Years in the last job | 0.99 | 0.95 | 1.03 | -0.01 | 0.02 | 0.65 |
| Member of a diabetes working group | 0.48 | 0.18 | 1.31 | -0.74 | 0.51 | 0.15 |
| Courses/professional education related to diabetes management in the last 12 months | 0.52 | 0.26 | 1.07 | -0.65 | 0.36 | 0.08 |
| Participation in clinical trials for diabetes or other metabolic/cardiovascular/renal diseases in the last 12 months | 1.92 | 0.90 | 4.07 | 0.65 | 0.39 | 0.09 |
| Workplace, ref: Urban  Semi-urban (1)  Rural (2) |  |  |  |  |  |  |
| Number approx. of patients in the quota | 1.22 | 0.55 | 2.68 | 0.20 | 0.40 | 0.63 |
| Number approx. of people with diabetes in the quota | 1.07 | 0.42 | 2.71 | 0.07 | 0.47 | 0.89 |
| Number approx. of patients with T2DM who visit weekly | 1.00 | 1.00 | 1.00 | 0.00 | 0.00 | 0.05 |
| Time approx. T2DM consultation per patient (min). | 1.00 | 1.00 | 1.00 | 0.00 | 0.00 | 0.62 |
| Male professional, ref: female | 1.00 | 1.00 | 1.01 | 0.00 | 0.00 | 0.39 |

CPG: clinical practice guidelines; T2DM: type 2 diabetes mellitus; CI: confidence intervals
